# Artificial amniotic fluid for nuclear magnetic resonance spectroscopy studies

**DOI:** 10.1002/ansa.202100055

**Published:** 2022-04-24

**Authors:** Doshina Naila, Siddharth Sadanand, Dafna Sussman

**Affiliations:** ^1^ Department of Electrical, Computer, and Biomedical Engineering Ryerson University Toronto Canada; ^2^ Institute for Biomedical Engineering Science and Technology (iBEST) at Ryerson University and St. Michael's Hospital Toronto Canada; ^3^ Department of Biomedical Physics Ryerson University Toronto Canada; ^4^ The Keenan Research Centre for Biomedical Science St. Michael's Hospital Toronto Canada; ^5^ Department of Obstetrics and Gynecology Faculty of Medicine University of Toronto Toronto Canada

**Keywords:** amniotic fluid, biomarkers, disease detection, non‐invasive, nuclear magnetic resonance imaging

## Abstract

Amniocentesis is the process of retrieving the nutrient‐rich amniotic fluid (AF) that encompasses the growing fetus in order to diagnose fetal diseases and developmental disorders. Currently, it is only performed on pregnant persons at risk and is invasive with the potential for infection and in some cases, miscarriage. A non‐invasive alternative is needed and could be developed using magnetic resonance spectroscopy (MRS). To develop such MRS sequences, ample testing and training are needed and could be most efficiently conducted on a phantom. We propose a protocol for creating such a synthetic AF phantom for MRS testing and optimization. The proposed AF is validated using nuclear magnetic resonance (NMR) proving it produces spectra comparable to those in the literature. The results from this study can aid in developing a non‐invasive fetal diagnostic tool to replace amniocentesis.

**Abbreviations**
1H‐NMR: proton nuclear magnetic resonance
AF: amniotic fluid
DI: deionized
FASTMAP: fast, automated shimming technique by mapping along projections
HMDB: human metabolomics database
LOCMAT: localized magic angle turning
LOQ: limit of quantification
MRI: magnetic resonance imaging
MRS: magnetic resonance spectroscopy
MRSI: magnetic resonance spectroscopy imaging
NMR: nuclear magnetic resonance

## 1 INTRODUCTION

The study of metabolomics allows for a snap image understanding of metabolic processes due to physiological, environmental, and genetic stimuli. Unlike transcriptomic and genomic studies, which uncover the genetic pathways underlying a biological process,^1^ metabolomic data are representative of the immediate short term biological state, providing better insight into the progression of the disease or disorders.^1^, ^2^, ^3^, ^4^, ^5^, ^6^

High resolution nuclear magnetic resonance (NMR) spectroscopy allows for the quantification of metabolites in ex‐vivo biological samples. Detection of metabolites with NMR can serve as a biomarker for the disease. This method can be applied to the study of amniotic fluid (AF), which surrounds the fetus during development, and provides essential nutrients and environmental conditions for optimal and healthy gestation.^7^, ^8^, ^9^, ^10^, ^11^ Amniocentesis is an invasive trans‐abdominal procedure administered to pregnant individuals with risk factors such as advanced maternal age, predisposition to genetic defects, family history of chromosomal abnormalities, autosomal recessive disorders, or potential pathologies on ultrasound. Previous studies have highlighted the importance of amniocentesis in diagnosing disease and potential risk factors in pregnancies.^7^ AF extracted through amniocentesis is analyzed to identify biomarkers that can affect the health of the fetus, and impact the decision to continue, intervene or terminate the pregnancy.^6^ Analysis of AF samples procured via amniocentesis can be done with NMR to further identify novel biomarkers of disease. However, amniocentesis causes stress to expectant mothers undergoing the procedure and can pose complications in pregnancy, such as miscarriage or risk of infection. One emerging platform for non‐invasive clinical metabolomics is the use of magnetic resonance spectroscopy (MRS) for the identification of biomarkers of disease. MRS builds on NMR by enabling the acquisition of tissue spectra without the need of removing tissue from the subject, by using clinical magnetic resonance imaging (MRI) hardware. Advances in MRS have allowed for the analysis of cells and tissue in vivo, permitting non‐invasive means of diagnosing disease and identifying genetic anomalies in gestation.^12^, ^13^, ^14^, ^15^, ^16^ Applying this technology to the metabolomic analysis of AF could replace invasive amniocentesis throughout gestation, and provide novel means of prenatal screening.^13^, ^14^, ^17^, ^18^ At present, however, spontaneous fetal motion, maternal respiratory and peristaltic motion, spectral shifts due to magnetic susceptibility interfaces, and large imaging volumes over which magnetic field strength can drift have hampered the application of the relatively slow MRS and MRS imaging (MRSI) pulse sequences to in vivo gestational imaging. Development of MRS and MRSI sequences appropriate for gestational imaging requires extensive imaging time and optimization that is not feasible with human subjects. Such development is the time‐limiting and financially‐constraining stage. The use of an artificial AF phantom in the context of a whole‐gravid uterus phantom in place of human subjects would then make the development of MRS pulse sequences for gestational imaging more accessible. Such a phantom would then permit not only extensive repeated testing but the ability to test a variety of imaging scenarios and to evaluate the reproducibility of the results. Spectroscopic studies have determined that AF metabolite concentrations correlate with gestational milestones, and are indicative of fetal metabolism, heart health, birth weight and maternal health as well. These analyses further indicated specific metabolites of interest in the case of aneuploidies, preeclampsia or spina bifida, to name a few.^13^, ^14^, ^17^ The missing piece is then a protocol for the production of artificial AF.

This paper aims to devise and evaluate a protocol for making an artificial AF MRS phantom emulating a third‐trimester uncomplicated pregnancy, facilitating the development of MRS‐based diagnostic methods and pulse sequence development. Since data on AF constituents in late pregnancy and at term are most readily available, these were used to devise a protocol for producing artificial AF. To achieve this, we first conducted a literature review to determine the expected constituents of healthy, third trimester AF, as found through prior NMR studies on AF collected postnatally, or through amniocentesis. Literature concentrations of AF constituents were averaged. We then found the physiological pH, and osmolality required to best mimic the conditions of the environment in‐utero at this time point. We then formulated an artificial AF and compared the NMR spectrum of our sample with those of normal, healthy, late‐gestation/term AF. This comparison was intended as a validation step to confirm the suitability of our protocol for emulating biological AF NMR properties.

## 2 METHODS

### 2.1 Data source and search strategy for AF composition

A thorough literature review was conducted using PubMed, Google Scholar, and Ryerson University Library databases to determine significant AF metabolites and their concentrations in solution. An initial search on these databases for 'AF composition' was used, which was followed by secondary searches including keywords 'fetal'/’fetus’ AND 'NMR'/’Nuclear Magnetic Resonance Imaging’ AND 'pregnancy'/ 'pregnant' (Table 1). For each of the successive searches, papers which returned information on animals, such as mice, rabbits and sheep were excluded from the preliminary articles that were reviewed.

**TABLE 1:** Alternate keyword search terminology

Keyword	Alternative terminology
Amniotic	Amnio‐ and amniotic fluid
Fetus	Fetal, foetus, prenatal, pregnancy, pregnant, placenta, pre‐term and gestation
NMR	Nuclear magnetic resonance imaging, nuclear resonance, MRS, magnetic resonance imaging, proton imaging, NMR spectroscopy and H‐NMR
Pregnant	Pregnancy, prenatal, gestational period, gestation and labour

### 2.2 Inclusion criteria

Articles that were chosen included those which reported findings of human AF, specifically of the late gestational period (≥32 weeks). Furthermore, of these limited papers, only those which included quantitatively‐measured concentrations of AF metabolites were included in the data collection. Articles which only discussed specific metabolites and their effects in relation to specific diseases, or those which were not written in English were excluded from this study.

### 2.3 Literature search results

**FIGURE 1:**
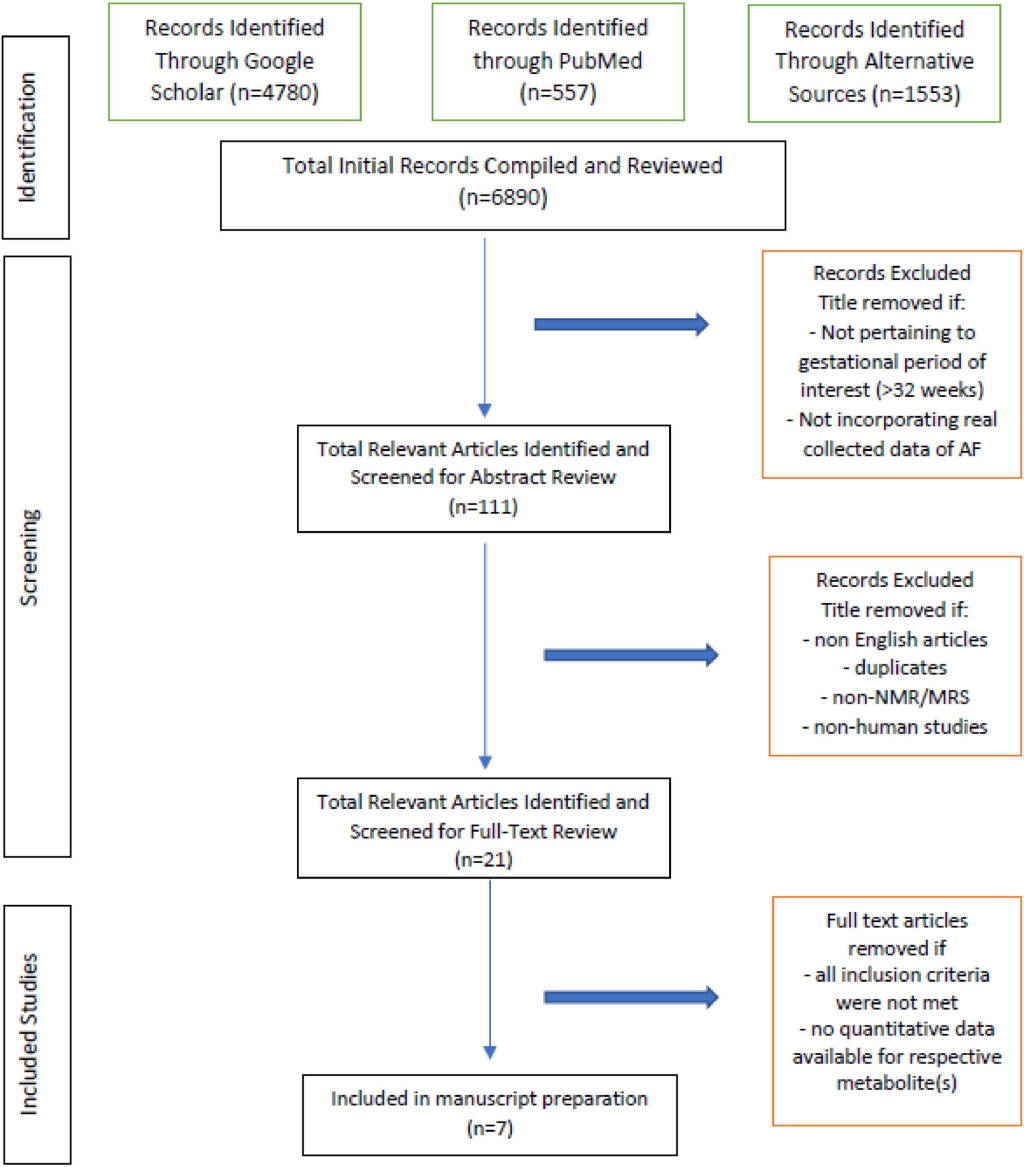
Flowchart of literature search and narrowing resources

An initial search by abstract and keywords 'Amniotic Fluid Composition' yielded 4759 results. Upon manual screening, articles were excluded if they were not pertaining to human AF, pertaining to imaging modalities other than NMR, and/or if they were non‐English or duplicate articles. Studies including metabolites constituent of AF, or discussion of pathogenesis, both without quantification of metabolite concentration were similarly excluded. This process is illustrated in Figure 1 which resulted in a total of seven papers that satisfied all selection criteria. AF metabolites from each of the articles were organized based on the article and the available metabolites, as well as their standard deviations (Table 2). These data were then converted into common units (umol/L), and compared in terms of concentration between the available papers. Metabolites which appeared only in one article were disregarded, as there was not enough supporting data to corroborate their inclusion nor confirm their measured concentrations. Statistical inconsistencies in the remaining metabolite concentrations were removed by excluding those data which fell outside the interquartile range. The extreme maximum and minimum values of each metabolite were compiled, and any outlying concentrations were excluded from our calculations. The average concentration of each metabolite was calculated as determined from literature values. Metabolites with an average concentration less than 40 umol/L were excluded from the data since this falls below the expected limit of quantification (LOQ) for NMR.^16^, ^19^, ^20^ A final list of metabolites common to at least two of the articles of interest was compiled and is shown in Table 2.

**TABLE 2:** Some amniotic fluid metabolites and their concentrations as identified from literature search

**Study** *			**1**	**2**	**3**	**4**	**5**	**6**	**7**
**Sample size**			**135**	**50**	**10**	**16**	**40**	**16**	**8**
**Condition of pregnancy°**			**Rh**	**U**	**U**	**Rh**	**U**	**U**	**U**
**Metabolite**									
**alanine**	mean	umol/L	126		689	156	457	194	152
	s.d.		26			66	119		
**glycine**	mean	umol/L	73			139		185	152
	s.d		25			28			
**proline**	mean	umol/L	102			130		103	132
	s.d		17			58			
**valine**	mean	umol/L	49	323	198	54	168	67	44
	s.d		16			22	52		
**glutamine**	mean	umol/L	91	310				223	277
	s.d		30						

* Each study was numbered according to the order in which they were read and recorded.

°Condition of pregnancies from which amniotic fluid was collected in each study is indicated by either U (uncomplicated pregnancy), or Rh (Rh disease requiring early induction).

^1^ Lind T et al.^21^

^2^ Cohn BR et al.^22^

^3^ Wilson et al.^20^

^4^ Reid et al.^3^

^5^ Sims et al.^16^

^6^ Levy HL and Montag PP^23^

^7^ Cockburn et al.^24^

### 2.4 pH and solubility calculations

As the components of the artificial AF are predominantly water and water‐soluble amino acids, it is important to maintain the solubility and pH conditions closely to what is observed in vivo. This is to minimize the common‐ion effect and le Chatelier's principle from affecting solute concentrations, particularly in polyprotic acids, which can skew results in the NMR spectrum.^2^, ^25^ The dissociation equilibrium of each analyte in a volume of deionized (DI) water was calculated in isolation from the others, and the resultant concentrations of all ionic species were recorded. Subsequently, a new equilibrium for each analyte was found with these new initial solute concentrations, and the resultant new equilibrium concentrations of ionic species were recorded. This process was repeated for 10 generations until the intergenerational change in total proton concentration was less than 0.005% of the variance of the predicted equilibrium concentration. Conjugate bases of dissociated polyprotic acids were introduced as separate species to the calculation in later generations with their respective acid dissociation constants.

To achieve a target pH of 7.25 and osmolality of 225 mM, as found in AF through literature review,^1^, ^21^ an initial solution osmolality from all dissociated ionic species at equilibrium and an initial pH from the equilibrium proton concentration were calculated. The surplus of NaOH needed to sequester protons to reach the target pH was then found, and a new solution osmolality was recorded. Finally, an according amount of HCl and NaOH required to reach the target osmolality was calculated for the artificial AF.

The resultant masses of HCl and NaOH needed to attain the target pH and osmolality were added to the experimental procedure. This method was used in lieu of a phosphate‐buffered solution to better account for the perturbation to the buffer pH caused by the metabolites dissolved. In particular, the concentration of the diprotic lactic acid would move a 1X PBS solution outside the range of the buffer, and subsequent correction of pH with HCl or NaOH would then affect osmolality.

### 2.5 Experimental procedure

The composition of AF is roughly 98%–99% water (v/v), with the remainder being constituents of the cell.^1^ Dissolved inorganics such as sodium, chloride, and carbon dioxide (CO_2_) exist in concentrations that mimic the extracellular environment of the cell and make up half of the remaining 1%–2% of AF.^1^, ^2^, ^10^ Organic materials make up the rest, balanced with proteins and small concentrations of carbohydrates that vary over the gestational period.^13^ From the literature review conducted, 17 amino acids and simple sugars were identified.^1^, ^2^ In accordance with studies that determined osmolality and pH of AF, metabolites identified were mixed in solution to create a 100 ml artificial AF sample. Table 3 provides a summary of the metabolites and their physiological range of concentrations according to our literature search results.

**TABLE 3:** Metabolite concentrations and specifications in amniotic fluid

**Metabolite**	**Molecular weight (g/mol)**	**Concentration in AF (umol/L)** *	**Literature 1H chemical shift (ppm)** **	**pKa** **
Citrate	294.10	385.66	2.52 (d), 2.65 (d)	3.05, ‐4.2
Glucose	180.16	2293.33	3.23 (dd), 3.40 (m), 3.46 (m), 3.52 (dd), 3.72 (m), 3.82 (m), 3.89 (dd), 4.63 (d), 5.22 (d)	11.3, ‐3
Lactic acid	90.08	11,443.33	1.32 (d), 4.10 (q)	3.78, ‐3.7
Alanine	89.09	295.66	1.47 (d), 3.77 (q)	2.47, 9.48
Cystine	240.29	66.33	3.18 (dd), 3.38 (dd), 4.10 (dd)	1.56, 9.34
Ethanolamine	61.08	44	3.13 (d), 3.81 (d)	15.61, 9.55
Glutamic acid	147.13	41	2.04 (m), 2.12 (m), 2.34 (m), 3.75 (dd)	1.88, 9.54
Glycine	75.07	137.25	3.54 (s)	2.31, 9.24
Histidine	155.16	49	3.16 (dd), 3.23 (dd), 3.98 (dd), 7.09 (d), 7.90 (d)	1.85, 9.44
Lysine	146.19	105.25	1.46 (m), 1.71 (m), 1.89 (m), 3.02 (t), 3.74 (t)	2.74, 10.29
Proline	115.13	116.75	1.99 (m) 2.06 (m), 2.34 (m), 3.33 (dt), 3.41 (dt), 4.12 (dd)	1.94, 11.33
Glutamine	146.15	225.25	2.13 (m), 2.45 (m), 3.76 (t)	2.15, 9.31
Serine	105.09	76	3.82 (dd), 3.96 (m)	2.03, 8.93
Taurine	125.15	119	3.25 (t), 3.42 (t)	−1.5, 9.34
Threonine	119.12	118	1.32 (d), 3.57 (d) 4.24 (m)	2.21, 9.00
Valine	117.15	129	0.97 (d), 1.03 (d), 2.26 (m) 3.60 (d)	2.72, 9.60
Creatinine	113.12	96.35	3.03 (s), 4.05 (s)	9.21, 4.96

* Average calculated concentrations in human AF at > 32 weeks gestation.

** Data collected from the Human Metabolome Database (HMDB)^29^ ^.^

Initially, the calculated average concentrations of each metabolite were used to determine individual masses of each respective metabolite for a 100 ml sample of artificial AF. Metabolites whose mass required to prepare 100 ml artificial AF were less than LOQ of an analytical balance (1 mg) were serially mass‐diluted from concentrated stock preparation to reduce error. Once dilutions were created, each metabolite was then added to DI water with continuous stirring, allowing for complete dissolution.

Upon the addition of the final metabolite, an internal standard was added at a known concentration. Calcium formate (traceable certified reference material) was used as a quantitative internal standard and chemical shift reference. Internal standards are pure compounds that are unrelated to the analyte and contain the nucleus of interest while having resonance that does not overlap with those of the metabolites in the sample.^26^, ^27^ This standard's concentration can then be used to determine those of the sample analytes. The internal standard in this experiment was expected to resonate between 7.5 and 8.5 ppm, occurring away from the analytes, which cluster between 0 and 4.5 ppm. The pH of the artificial AF was then measured and titrated to the target pH using concentrated NaOH and HCl to establish physiological conditions. NaOH and HCl were then added in equal parts to attain the target osmolality (225 mM).^24^, ^28^ After producing the initial mixture of artificial AF, the sample was lyophilized and reconstituted in deuterated water, to better attenuate the solvent signal from overwhelming analyte peaks.

Glucose, an extremely important metabolite for various clinical applications also acts as an important AF biomarker for various diseases. In previous studies conducted,^2^, ^8^ glucose in AF and serum were used to determine the development of fetal abnormalities such as fetal hypoxia and energy metabolism. This metabolite is not only important but abundantly found across AF samples in the literature. Although glucose pervades various regions of the NMR spectra, it often overshadows other metabolites in the same vicinity along the spectrum. Previous AF characterization studies reported the presence of both alpha and beta glucose, which exist as isomers in solution, and can be detected distinctly on an NMR spectrum. To determine glucose concentrations with reference to literature, two separate experiments were run in tandem. The first included all metabolites and additives to the AF, as mentioned in the protocol, while the second omitted glucose only. The purpose of this experiment was to observe the variation in the spectra as a result of glucose addition.

### 2.6 MR spectroscopy

A standard 1H NMR (699.80 MHz) spectrum was acquired at 25°C using an Agilent DD2 spectrometer equipped with a 5 mm sample tube in a 1H‐19F {13C/15N} Triple Resonance Cold Probe. The 1H NMR was acquired with a pulse width of 9.3 μs (transmitter power of 53 db), 256 scans, a d1 delay of 10.1 s, and an acquisition time of 20.04 s totalling 455k points in the free induction decay.

### 2.7 Statistical analyses

All metabolites that comprised the artificial AF were analyzed amongst literature and experimental values to determine statistical equivalence (see appendix). To compare the concentration of metabolites in the artificial AF and literature derived values, a non‐parametric test was first conducted. The Mann‐Whitney U Test, also known as Mann Whitney Wilcoxon or Wilcoxon Rank Sum Test, was used to determine whether the two populations have the same distribution. This test was conducted at a significance of alpha = 0.05.

Upon determining the similarities in the spread of the data, a two‐sample paired t‐test was performed to determine whether these averages are statistically similar or significantly different from one another. The null hypothesis for this test states that both means are statistically similar, at α = 0.05.

Finally, upon comparing the means of the two separate groups, we conducted an analysis of variance (ANOVA) to determine the significance of variance in each metabolite in either group. Metabolites that were found to be statistically significantly different between the groups were further analyzed using *t*‐tests at α = 0.05.

## 3 RESULTS

**FIGURE 2:**
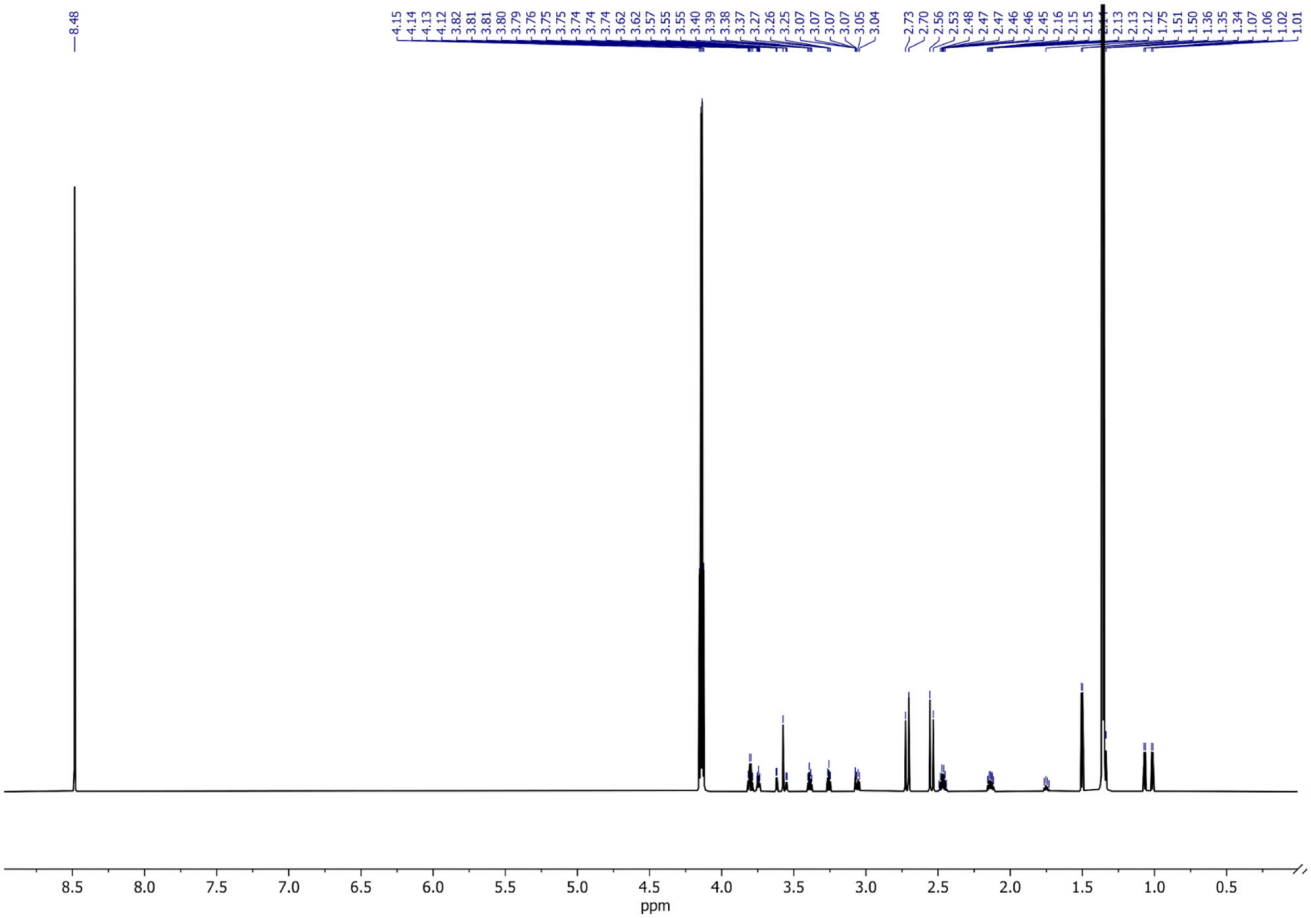
One‐dimensional 700 MHz proton nuclear magnetic resonance (^1^H NMR) spectrum of artificial amniotic fluid, after lyophilization and reconstitution in deuterated water (D_2_O)

**FIGURE 3:**
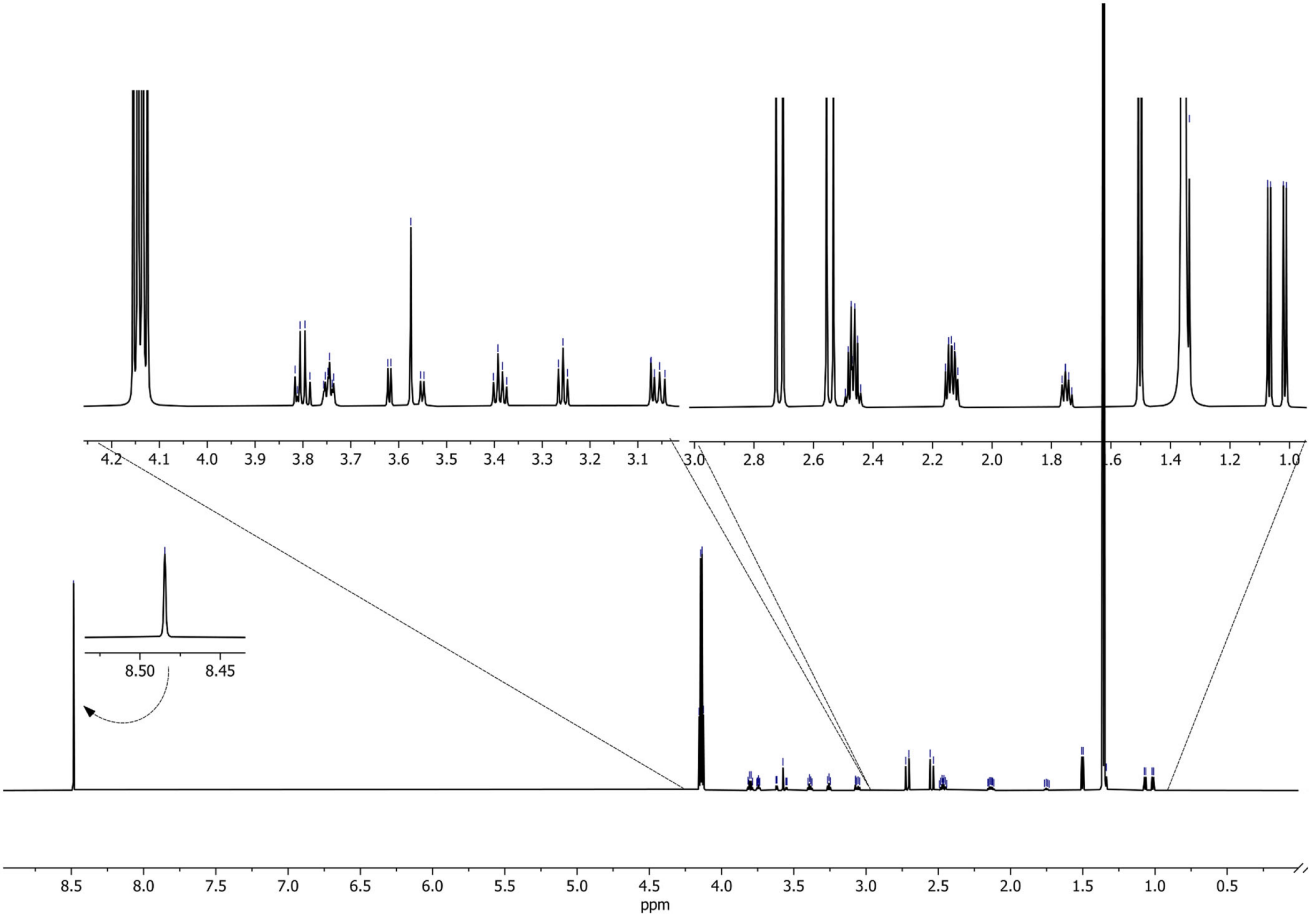
One‐dimensional 700 MHz proton nuclear magnetic resonance (^1^H NMR) spectrum of artificial amniotic fluid with magnifications at ranges across the spectra of interest

**FIGURE 4:**
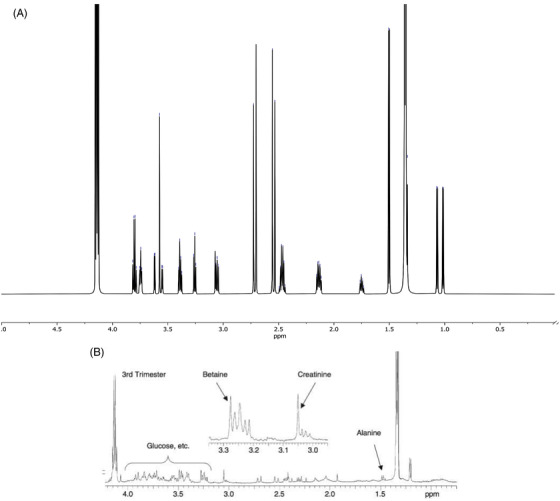
(A) One‐dimensional 700 MHz proton nuclear magnetic resonance (^1^H NMR) spectrum of artificial amniotic fluid from 0–5.0 ppm. (B) 1D 500 MHz 1H HR‐MAS spectrum of healthy amniotic fluid from the third trimester of an uncomplicated pregnancy. Figure reproduced with permission from MAGMA: Cohn, BR^21^

**FIGURE 5:**
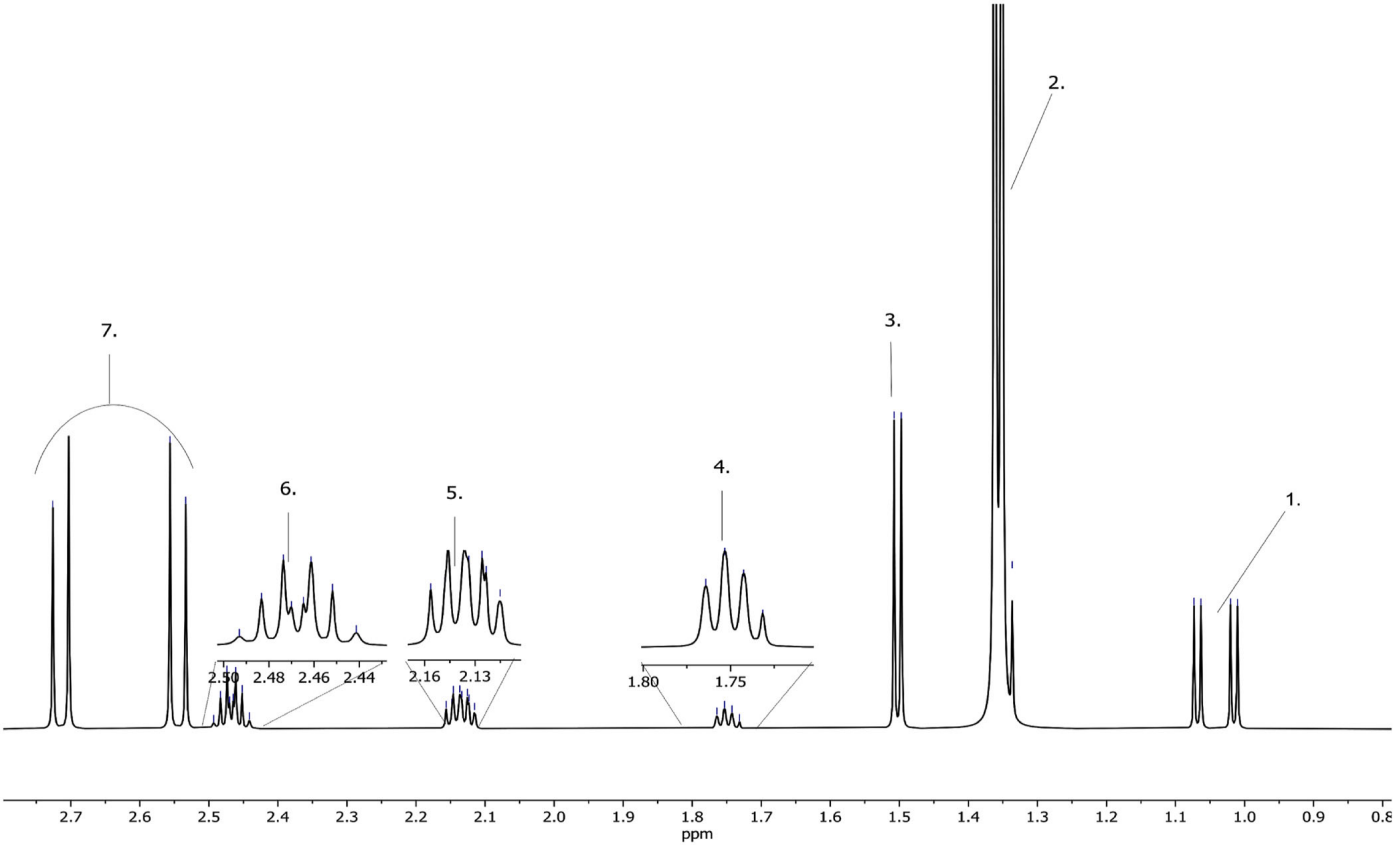
Magnified 1D proton nuclear magnetic resonance (^1^H NMR) spectrum showing metabolites of artificial amniotic fluid within ranges of 0.8–2.8 ppm. Assignments of peaks: 1. Valine, 2. Lactic Acid, 3. Alanine, 4. Lysine, 5. Proline, 6. Glutamine, 7. Citrate

**FIGURE 6:**
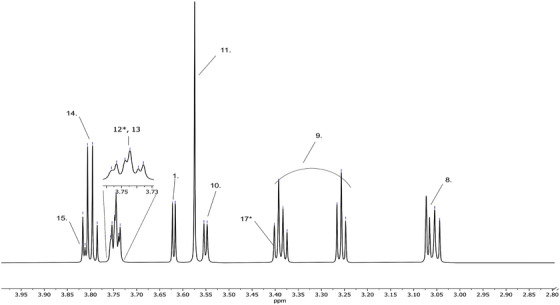
Magnified 1D proton nuclear magnetic resonance (^1^H NMR) spectrum showing metabolites of artificial amniotic fluid within ranges of 2.8–4.0 ppm. Assignments of peaks: 8. Cysteine, 9. Taurine, 10. Threonine, 11. Glycine, 12. Glutamic Acid**, 13. Ethanolamine, 14. Serine, 15. Creatinine, 17. Histidine**

**FIGURE 7:**
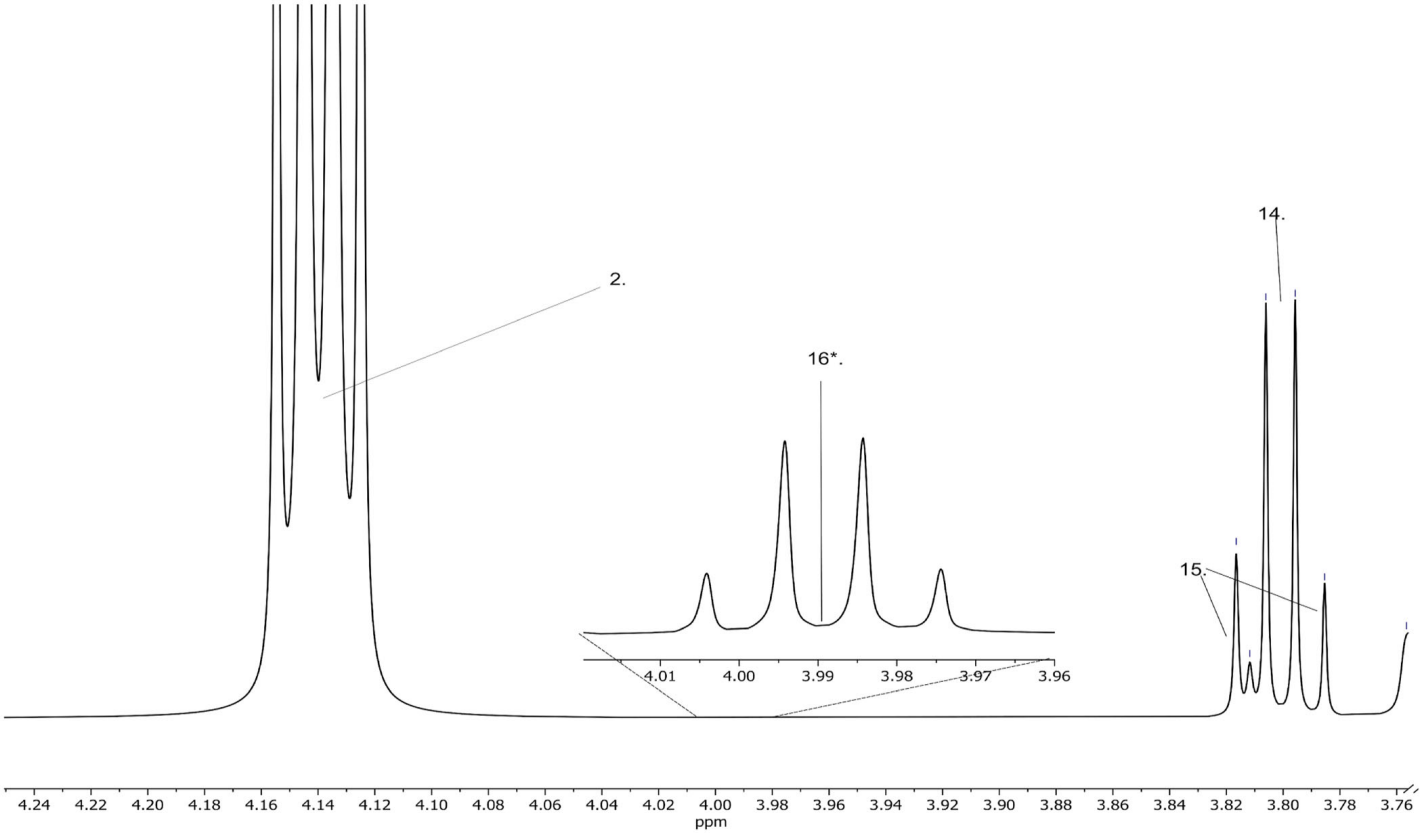
Magnified 1D proton nuclear magnetic resonance (^1^H NMR) spectrum showing metabolites of artificial amniotic fluid within ranges of 3.75–4.25 ppm. Assignment of peaks: 2. Lactic Acid, 14. Serine, 15. Creatinine, 16. Glucose **

**FIGURE 8:**
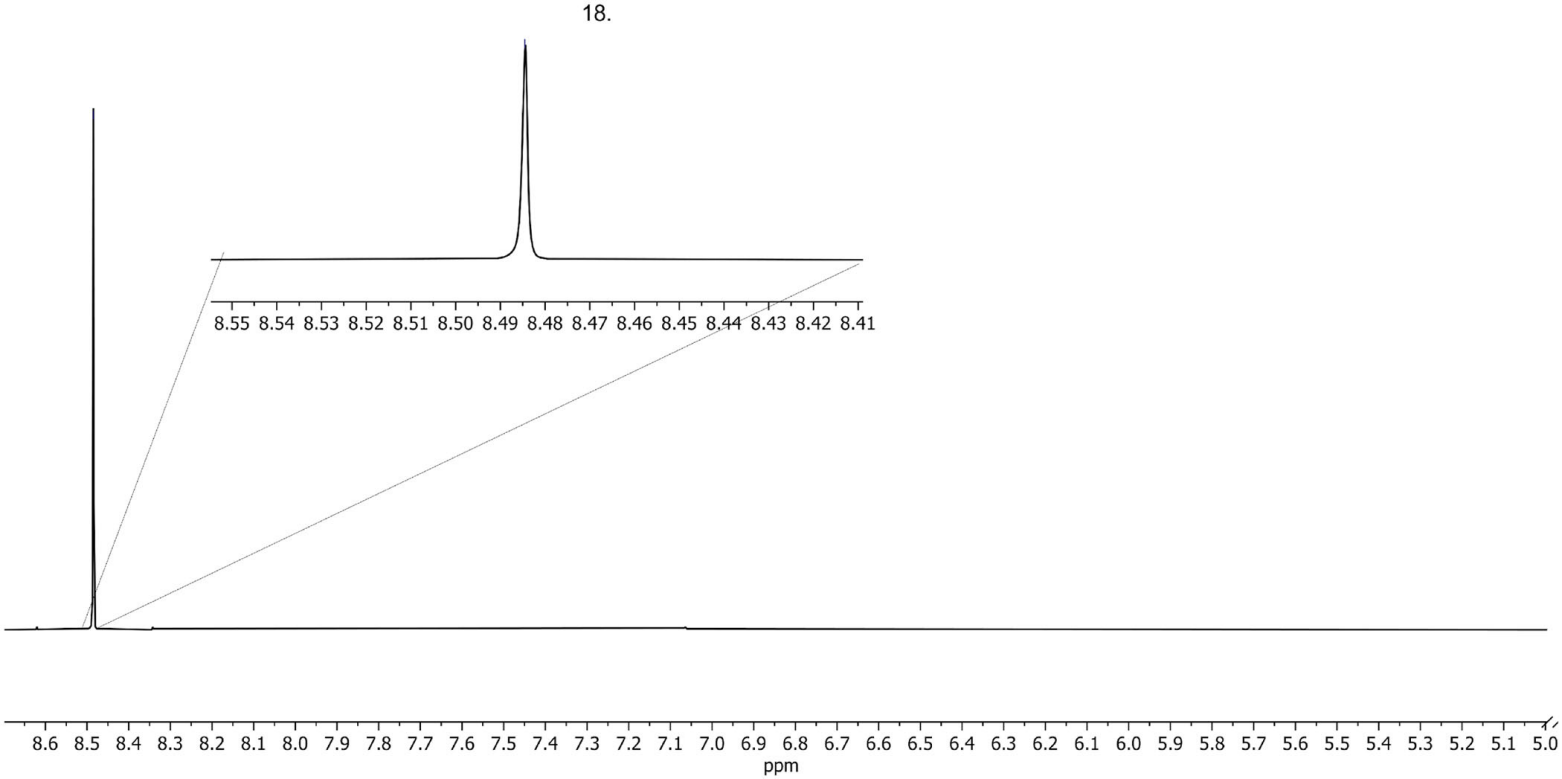
Magnified proton nuclear magnetic resonance (^1^H NMR) spectrum showing metabolites of artificial amniotic fluid within ranges of 5–8.7 ppm. Peak assignment: 18. Calcium Formate (quantitative nuclear magnetic resonance [qNMR] standard analyte)

The entire 1D proton NMR (1H‐NMR) spectrum of the artificial AF is shown in Figure 2, encapsulating 0–9 ppm to capture all the metabolites. Figures 3 and 4 depict magnifications of the spectral regions of interest. Figures 5, 6, 7, 8 focus on smaller ranges of the spectrum and identify the metabolites which are found in each range with peak assignments respective to each metabolite. '**' indicates overlapping peaks that cannot be quantified accurately in our analyses. Although integration of these metabolites were not possible, the respective peaks appear in the NMR spectra and are noted in the figures below. All peaks in the spectra are respectively identified using the Human Metabolomics Database (HMDB).

**FIGURE 9:**
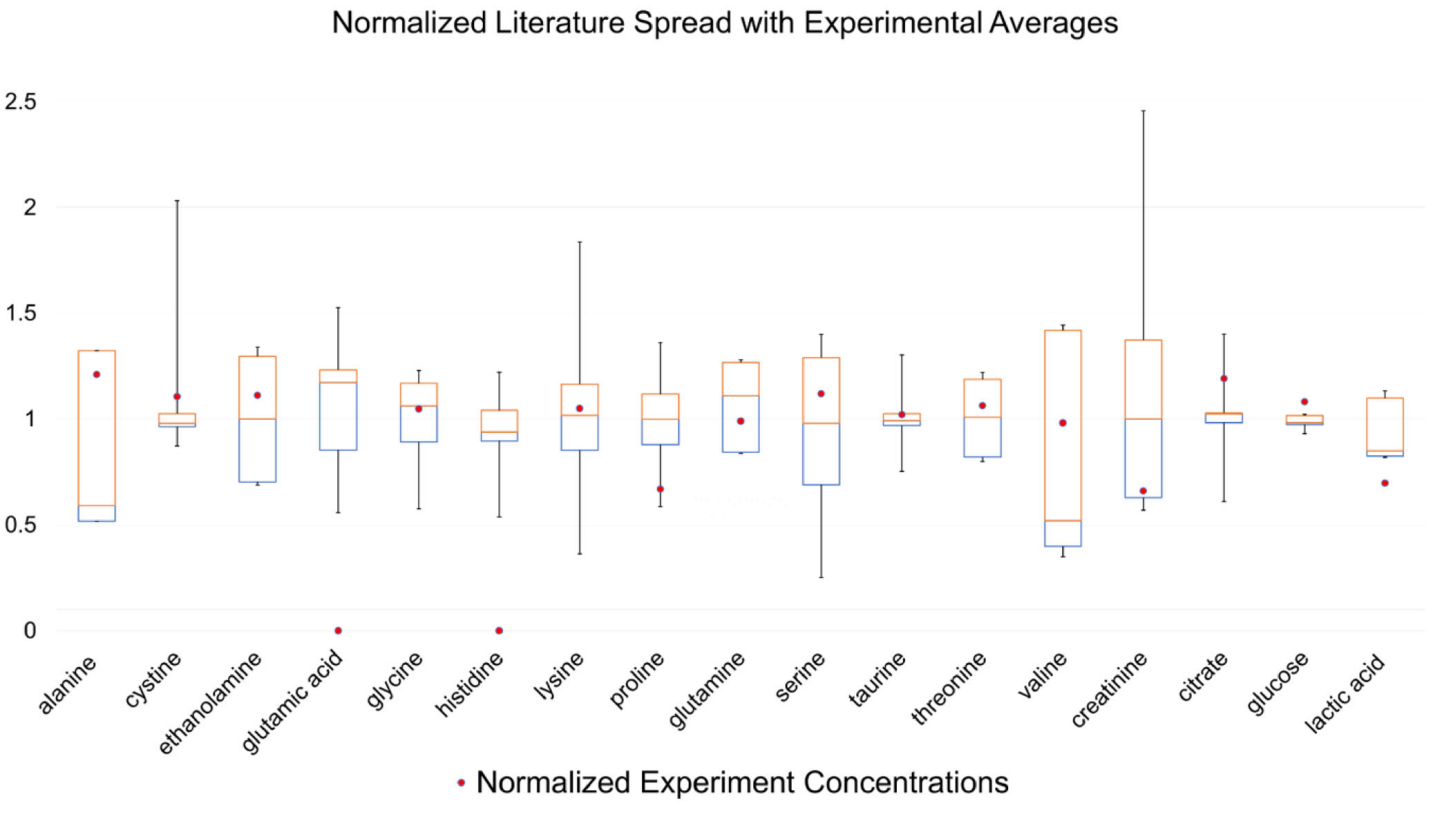
Normalized concentrations of literature data collected from studies utilized to create artificial amniotic fluid. Experimental concentration averages derived from nuclear magnetic resonance (NMR) analysis are shown using a 'dot' as they appear along the distribution of literature values. Artificial amniotic fluid (AF) concentrations of glutamic acid and histidine were not quantifiable and are, therefore, shown as 0 on this graph

The calculated concentrations of each metabolite in the artificial AF from NMR spectroscopy are compared with those found in the literature from equivalent NMR studies of biological AF. After normalizing the data, Figure 9 illustrates the differences between the observed concentrations for each metabolite used in the artificial AF.

**FIGURE 10:**
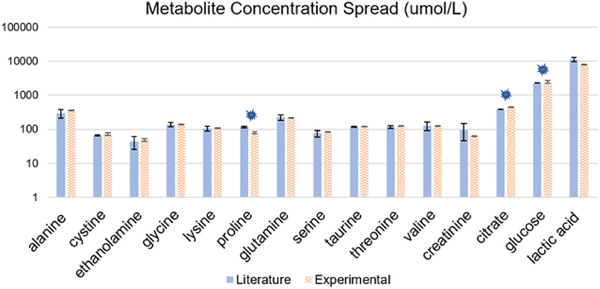
Metabolite concentrations in amniotic fluid as they appear in the literature (blue) versus the experiment (orange). (* *p* < 0.05)

**FIGURE 11:**
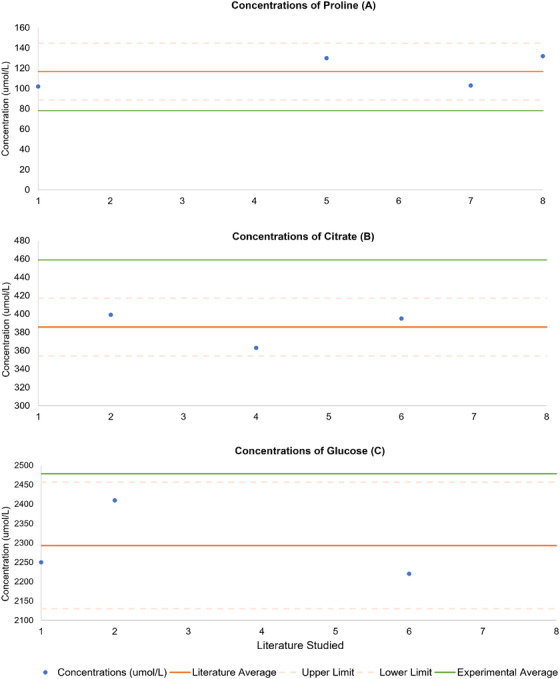
Bland Altman plot representing the concentrations (umol/L) of metabolites proline (A [*p* = 0.005, α = 0.05]), citrate (B [*p* = 0.007, α = 0.05]) and glucose (C [*p *= 0.050, α = 0.05]) in the literature. Normalized values are compared to those found experimentally

Proline, citrate and glucose yielded concentration results that were significantly different from anticipated literature values; (*p*_proline _= 0.005, *p*_citrate _= 0.007, *p*_glucose _= 0.050, at α = 0.05, Figure 10). These results were then further analyzed using an ANOVA and *t*‐tests to determine true differences in the data and are represented in Figure 11. Consulted literatures and their respective averages are also presented in the figures below. The various concentrations of respective metabolites are shown using a Bland‐Altman plot in Figure 11. Here, proline is studied across four different studies, and their normalized values are compared to those found experimentally. The concentration of proline in the artificial AF was found to be statistically different from literature values (*p* = 0.005, α = 0.05).

Glucose concentrations from the experiment were found to be significantly different from those in the literature (*p* = 0.050). This difference is attributed to alpha/beta isomerism confounding peak integration.^11^, ^30^ Although the significance of the comparison is weak, the distinction of these isomers in MRS imaging could prove diagnostic of fetal health and well being.

Similarly, proline and citrate were also found to be statistically different from their literature counterparts (*p*_proline _= 0.005 and *p*_citrate _= 0.007). Both metabolites fall within heavily congested areas of the NMR spectrum, with proline at 1.99, 2.06, 2.34, 3.33, 3.41 and 4.12 ppm; and citrate at 2.52 and 2.65 ppm (Table 3). Both metabolites heavily overlap other metabolites in this region, which can contribute to error.

## 4 DISCUSSION

A protocol for making an artificial AF phantom was created based on the anticipated ranges of concentrations of metabolites in biological AF as identified in the literature and quantified via 1H‐NMR. The protocol was then tested by comparing its 1H‐NMR spectrum against the literature in terms of the anticipated spectral peaks, splitting patterns, and peak integration.

Metabolites were identified and integrated within the chemical shift ranges reported in the HMDB, and are consistent with the spectra found in the literature review. Fifteen of the 17 metabolites in the artificial AF were able to be identified by 1H‐NMR spectrum peak chemical shifts and splitting patterns consistent with their respective structures (depicted in appendix Figures 12–23). The concentrations of 12 of these metabolites were statistically similar to their concentrations in biological AF found through the literature review. Peaks for histidine and glutamic acid were unable to be identified, as they were obscured by peaks of metabolites in higher concentration in the crowded aliphatic spectral regions of 2.0–2.8 and 3.1–3.8 ppm. Concentrations of glucose, citrate and proline in the artificial AF were however statistically different from the literature values (*p* = 0.050, *p* = 0.007 and *p* = 0.005, respectively).

Our findings for citrate are consistent with existing literature,^31^ which suggests that reproducibility of the metabolite in NMR is not always accurate. Citrate is often discussed as a significant metabolite, especially in reference to the citric acid cycle. In a study conducted to analyze the NMR peak reproducibility of citrate, it was found that NMR signals often doubled or disappear completely.^31^ Although no specific references were made to AF characterisation, these studies analyzed human blood and urine samples and their respective NMR spectra. This phenomenon is not well understood and has been attributed by Ross et. al. to be due to the sensitivity of citrate to partial deprotonation in the biological pH range of 4–8.^31^, ^32^, ^33^ Consistent with literature then, citrate should not be used as the sole determinant of diagnosis or detection of diseases.^31^, ^33^

### 4.1 Overview of artificial AF in practice

Spectroscopic studies have determined that AF metabolite concentrations correlate with gestational milestones.^13^ Renal function can be assessed using a combination of several metabolite concentrations, such as indoxyl‐sulphate, histidine, and formate.^5^, ^20^, ^21^, ^22^ Similarly, fetal metabolism, heart health, birth weight, and implications on maternal health, such as diabetes, have correlated with varying concentrations of amino acids found in AF.^8^, ^25^, ^34^ Overall, spectroscopic analysis of AF is a promising fetal diagnostic tool that could also be used to identify biomarkers of aneuploidies, preeclampsia, preterm delivery, spina bifida and low birth weight, to name a few.^2^, ^5^, ^6^, ^10^, ^11^, ^18^, ^22^, ^26^, ^28^, ^34^, ^35^ The ability to simulate AF will provide researchers with a tool to facilitate the development of non‐invasive metabolic MR‐based fetal diagnostic tools to determine causes and correlations of disease with various gestational metabolites.

Emerging MR contrast mechanisms and hardware, such as saturation transfer and high clinical fields (≥7 T), are enabling more specific and sensitive detection of metabolites through spectroscopic MRI. Higher clinical fields resulting in narrower linewidths allow for increased specificity and separation of confounds in spectroscopic imaging, while sequences such as steady‐state chemical exchange saturation transfer amplify metabolite signal, allowing quantification in milli‐ and micromolar concentrations. Such sequences and hardware would be well‐applied in gestational imaging, but there is a need to adapt these sequences to address the challenges of the gestational setting: motion and field inhomogeneity insensitivity.

In this study, we used literature data on AF constituents close to or at birth to create a protocol for fabricating synthetic AF. Given that amino acid concentration does not change significantly between the 6th and 9th months of gestation,^23^ the established protocol can be used to synthesize third trimester AF, in general. Such a phantom would be useful in constructing a whole‐gravid uterus phantom for the development of metabolic MRI pulse sequences to identify biomarkers of disease.

### 4.2 Recommendations and limitations

This paper exclusively explored producing an artificial AF phantom for MRS pulse sequence development and validated its similarity to biological AF using 1H‐NMR spectroscopy. NMR was used for validation due to its higher spectral resolution, sensitivity, and comparability to literature spectra of biological AF compared to MRS techniques. Current in‐vivo MRS methods done on intact live species often produce increased line widths, confounding metabolite quantification. These increased linewidths significantly limit the applicability of MRS and other modes of metabolomic imaging.

Additionally, further improvements in MRS signal acquisition, reduction of voxel size, or the application of high order shimming could enable MRS for use in clinical applications. Shimming is done to homogenize the static magnetic field and is often used to produce high‐quality NMR and MRS measurements.^36^ There are a variety of shimming methods and procedures, an example being FASTMAP (fast, automated shimming technique by mapping along projections). This approach has been demonstrated in the liver, muscle and septum of the heart of a live mouse, and humans.^37^ Another promising technique, termed LOCMAT (localized magic angle turning) has been used in previous studies on the liver and heart of a live mouse at relatively low magnetic fields (2 T). As discussed in a paper by Wind, Hu and Majors, this process was successful in analyzing areas in the body at low fields, and they hypothesize even better resolution improvements with higher magnetic fields.^25^

The limitations of this study are within the scope of the existing literature on AF and its constituents, their concentrations, and their presence in the late gestational period. Further data must be collected from patient samples throughout gestation in order to develop accurate phantoms representative of early gestation. Using such a phantom to develop MRS and MRSI pulse sequences, researchers can further their understanding of disease detection and treatment well before birth.^25^, ^38^ Furthermore, expanding the data on AF constituents across various time points in pregnancy will allow for more accurate anthropomorphic AF phantoms throughout gestation. A current state‐of‐the‐art solution could involve the use of artificial intelligence in determining spectral similarities, decreasing the chance of human errors, such as the program developed by Castillo et al.^39^ These technological advances can also be optimized to mute surrounding noise from tissues. In the future, higher field strengths, greater sampling bandwidth, or lesser sampling noise could potentially help decrease the margins of error and thereby give even more accurate and reproducible measurements as required in the detection of biomarkers of disease.

## 5 CONCLUSIONS

This study presents the first detailed protocol for synthesizing artificial AF for late gestation and compares the resultantly acquired 1H‐NMR spectrum against available literature. Being able to synthesize an artificial AF phantom will facilitate and expedite the technical development of metabolomic MRS sequences. In vivo, AF MRS holds promise in permitting non‐invasive detection of gestational disease, and in facilitating understanding of the correlations between AF constituents and fetal development and health.

## AUTHOR CONTRIBUTIONS

Doshina Naila: Conceptualization, methodology, formal analysis, investigation and writing

Siddharth Sadanand: Methodology, validation, formal analysis and writing – review and editing

Dafna Sussman: Conceptualization, resources, supervision, funding and writing – review and editing

## CONFLICT OF INTEREST

The authors have declared no conflict of interest.

## Supporting information

Supporting information

## Data Availability

Data sharing is not applicable to this article as no new data were created or analyzed in this study.
