# Supplementary material for: Artificial amniotic fluid for nuclear magnetic resonance spectroscopy studies
Source: Anal Sci Adv. 2022 Apr 24;3(5-6):174–87. doi: 10.1002/ansa.202100055 (PMC10989604; doi:10.1002/ansa.202100055)
Supplement: Supplementary file 1 — Supporting information [file ANSA-3-174-s001.docx]

### Supplementary Material


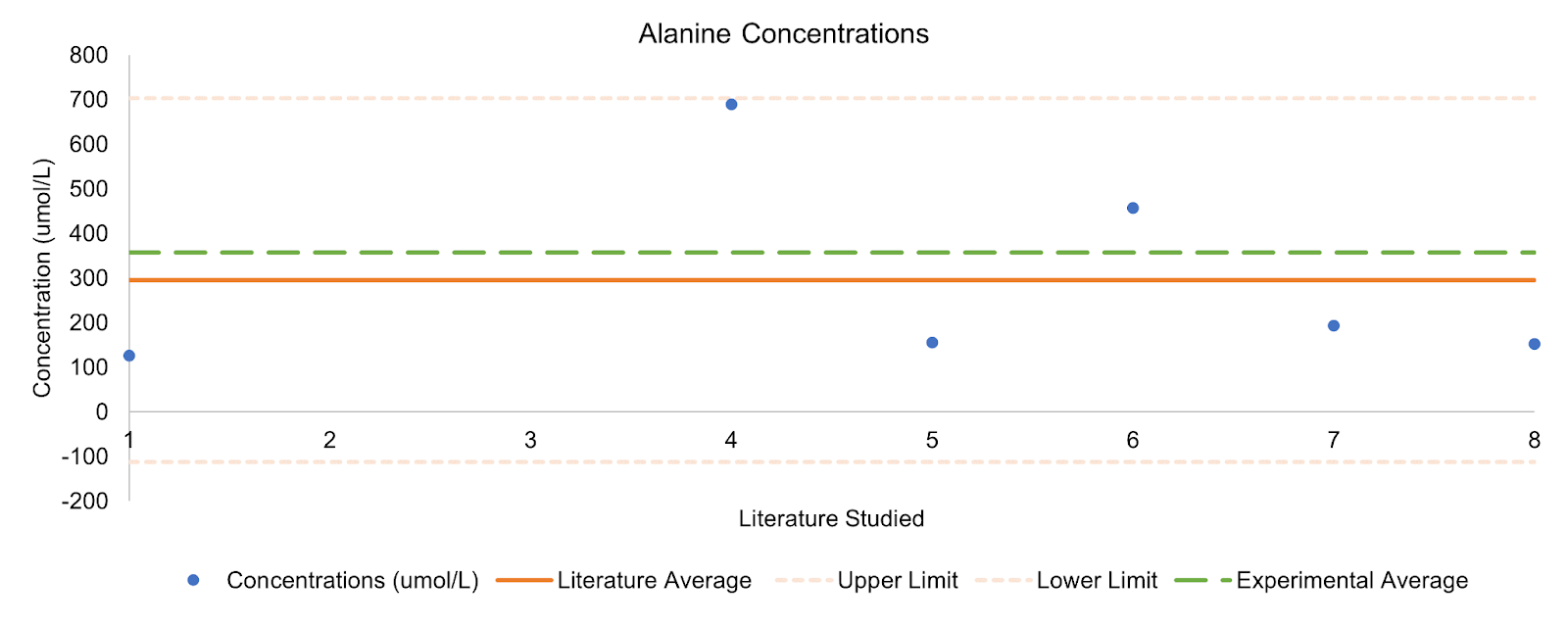


**Figure 12** The distribution of alanine concentrations as they appear in the literature and through experimental values. Experimental values and literature averages are statistically equivalent (p=0.34, a=0.05)


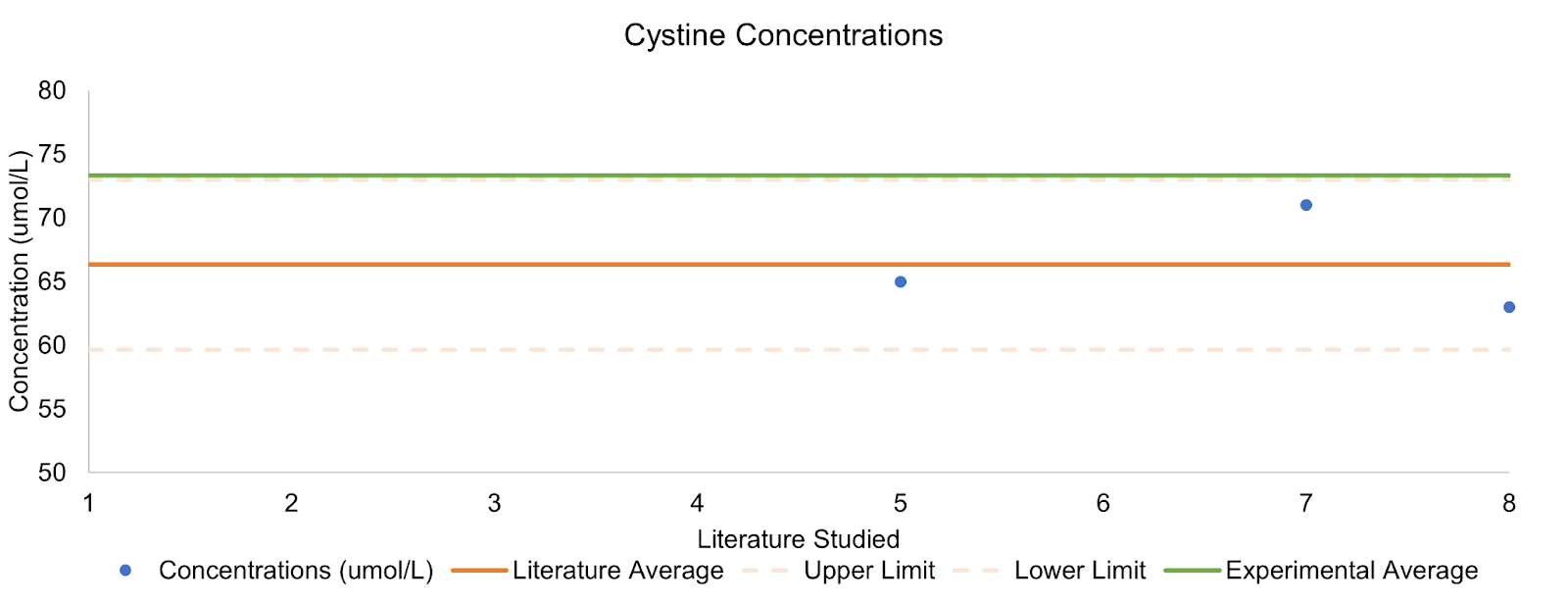


**Figure 13** The distribution of cystine concentrations as they appear in the literature and through experimental values. Experimental values and literature averages are statistically equivalent (p=0.06, a=0.05)


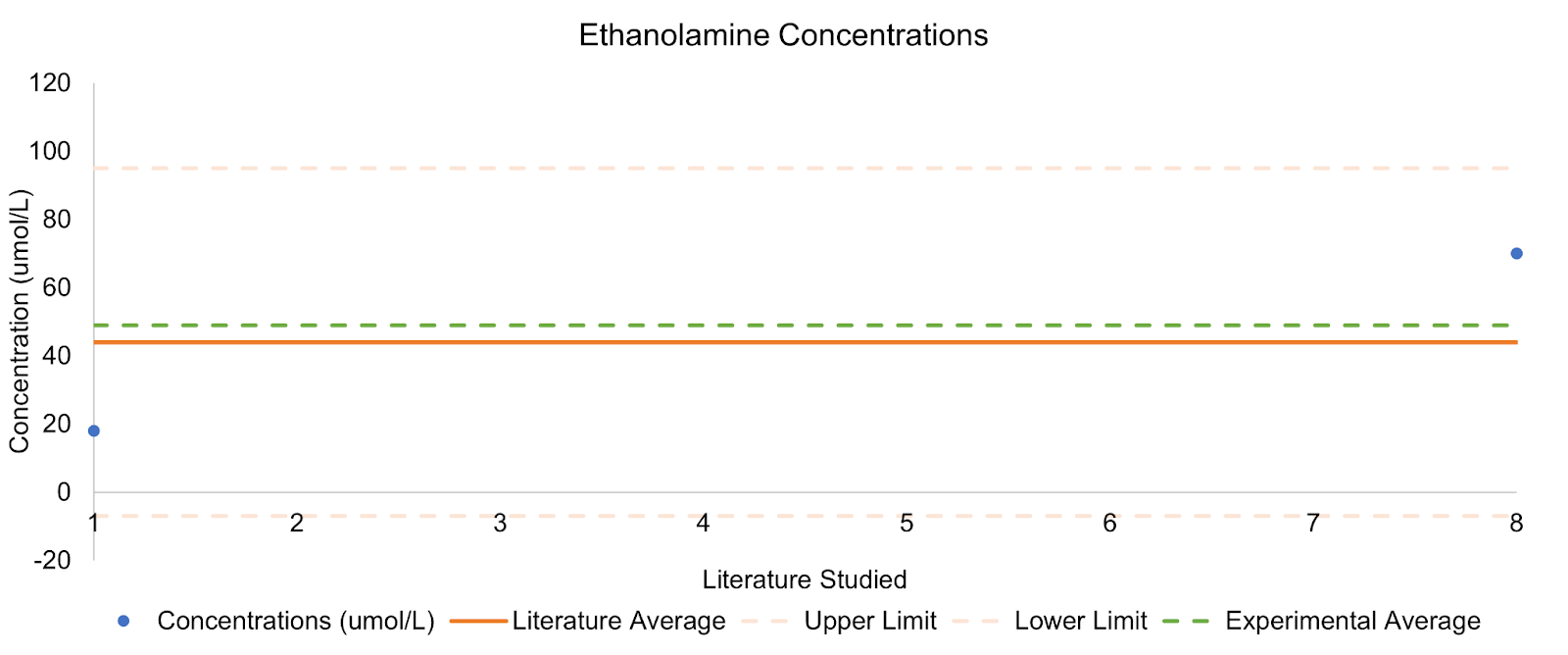


**Figure 14** The distribution of ethanolamine concentrations as they appear in the literature and through experimental values. Experimental values and literature averages are statistically equivalent (p=0.46, a=0.05)


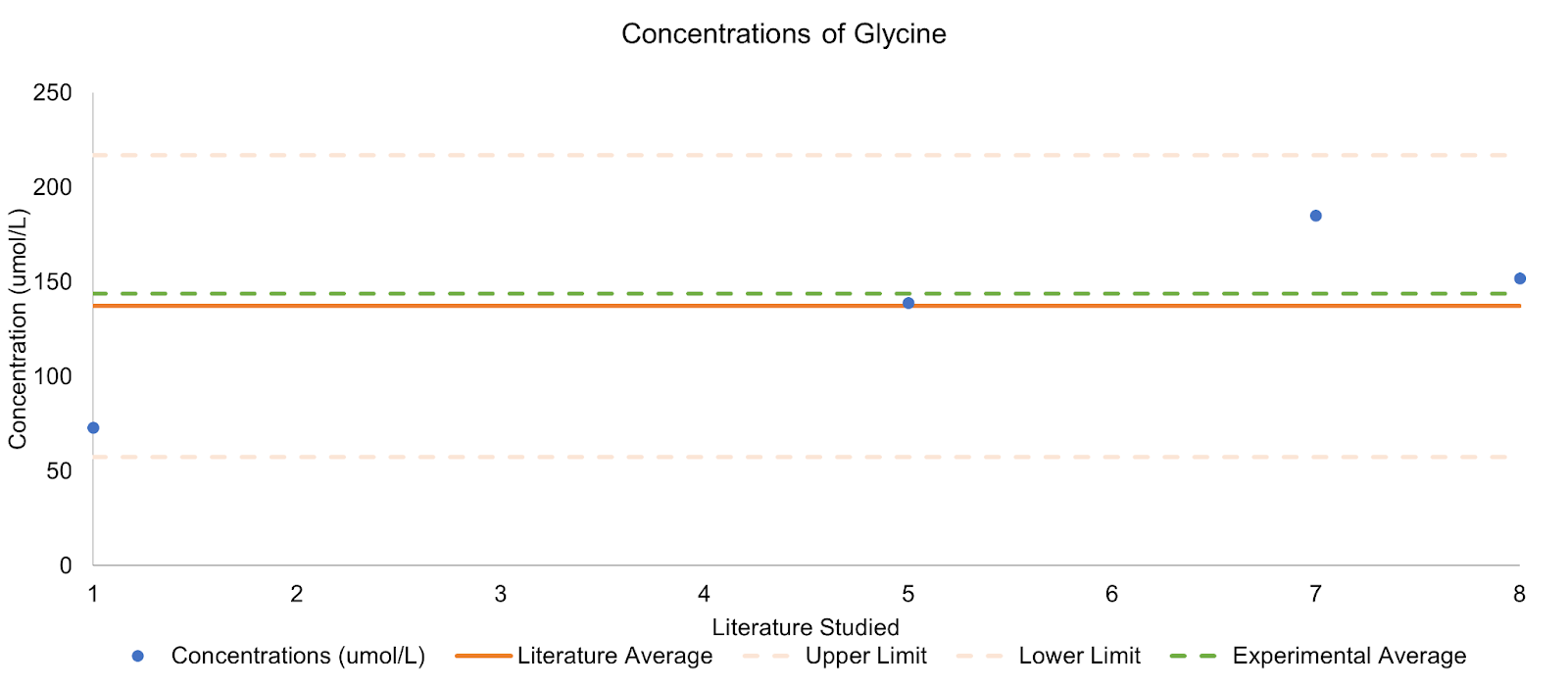


**Figure 15** The distribution of glycine concentrations as they appear in the literature and through experimental values. Experimental values and literature averages are statistically equivalent (p=0.43, a=0.05)


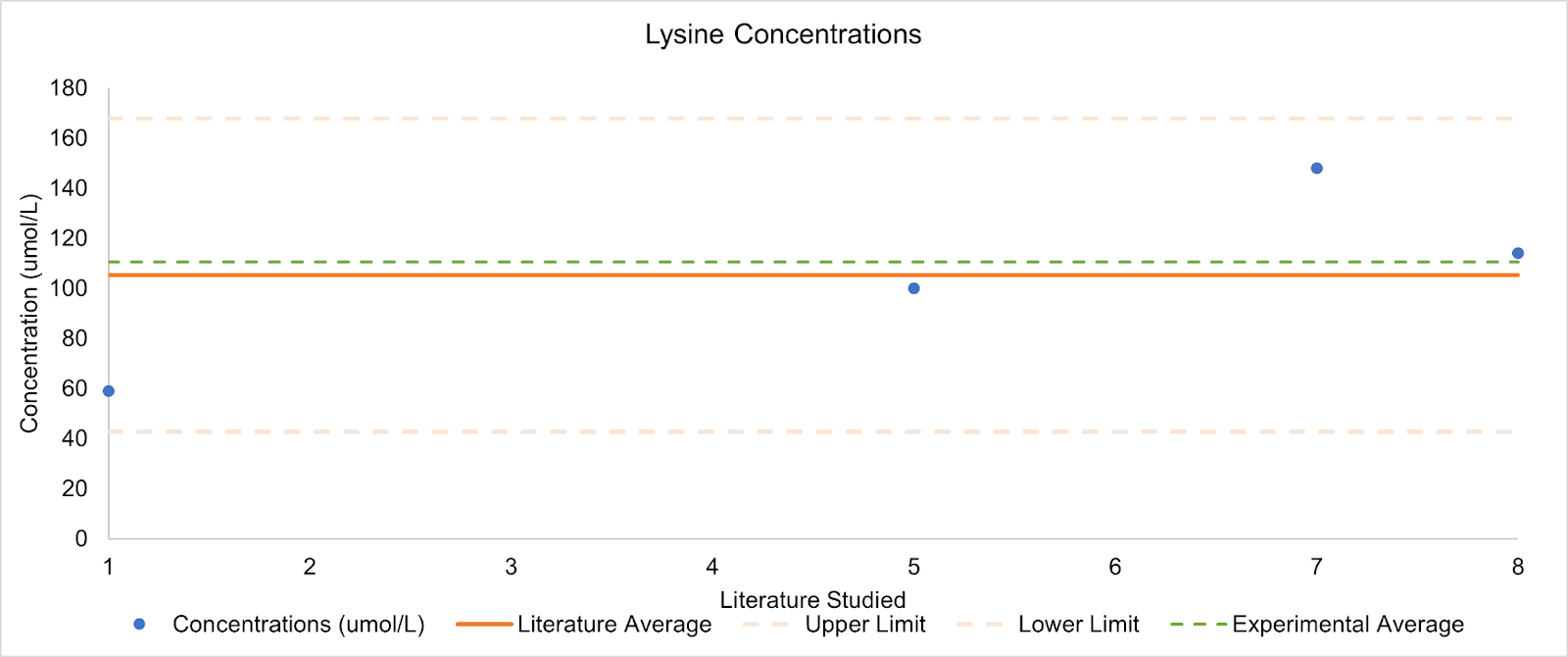


**Figure 16** The distribution of lysine concentrations as they appear in the literature and through experimental values. Experimental values and literature averages are statistically equivalent (p=0.43, a=0.05)


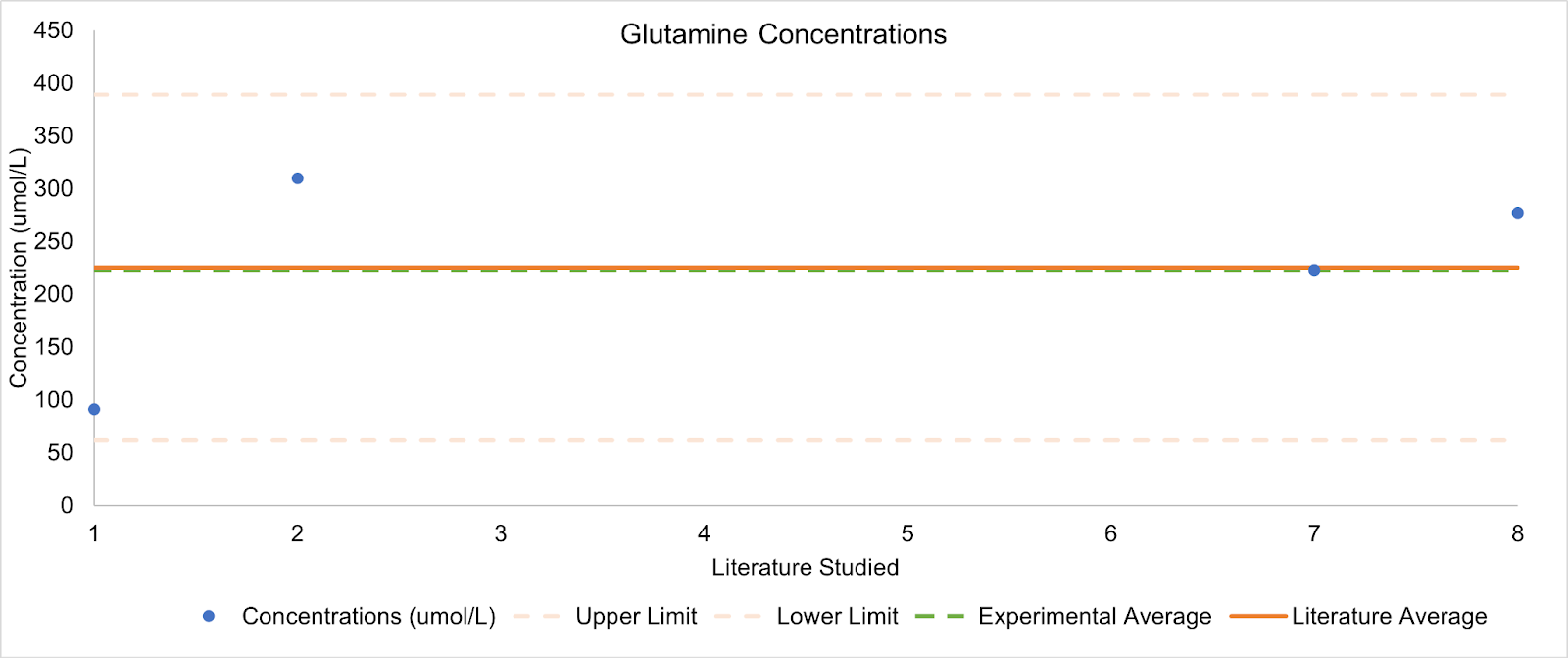


**Figure 17** The distribution of glutamine concentrations as they appear in the literature and through experimental values. Experimental values and literature averages are statistically equivalent (p=0.49, a=0.05)


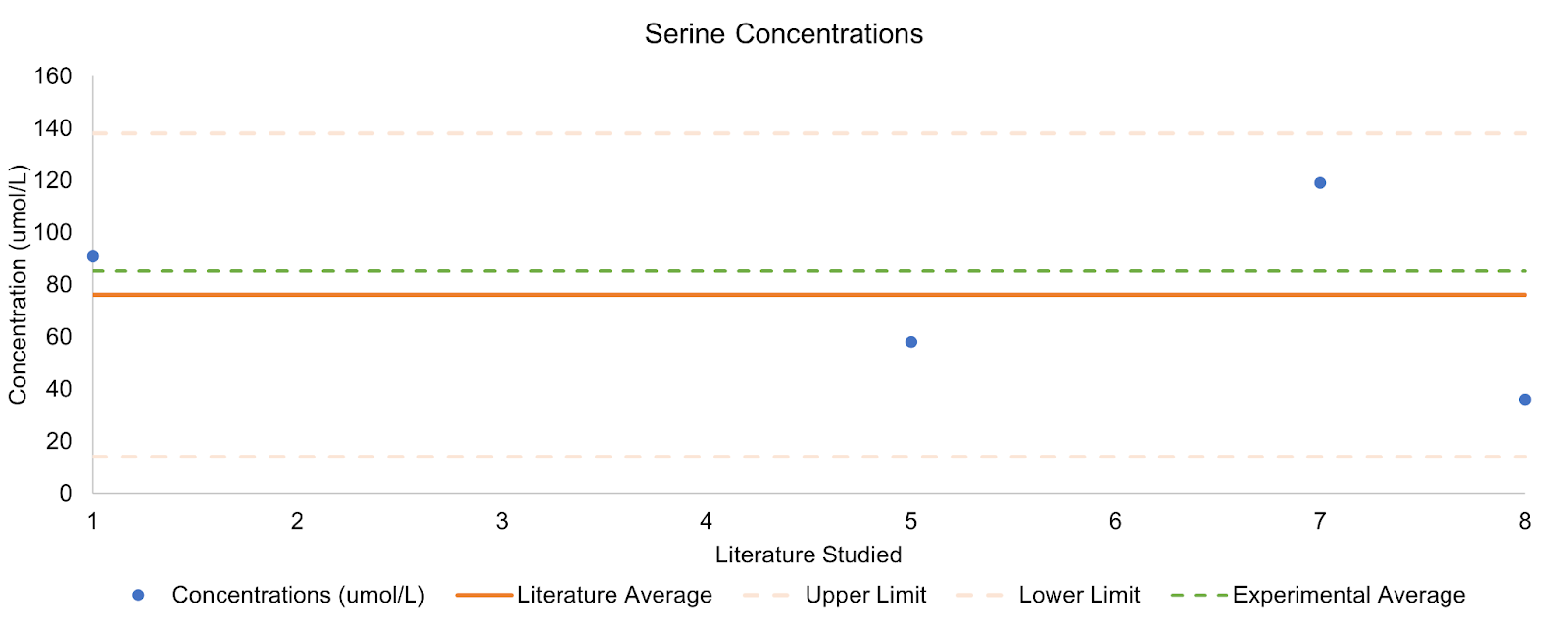


**Figure 18** The distribution of serine concentrations as they appear in the literature and through experimental values. Experimental values and literature averages are statistically equivalent (p=0.38, a=0.05)


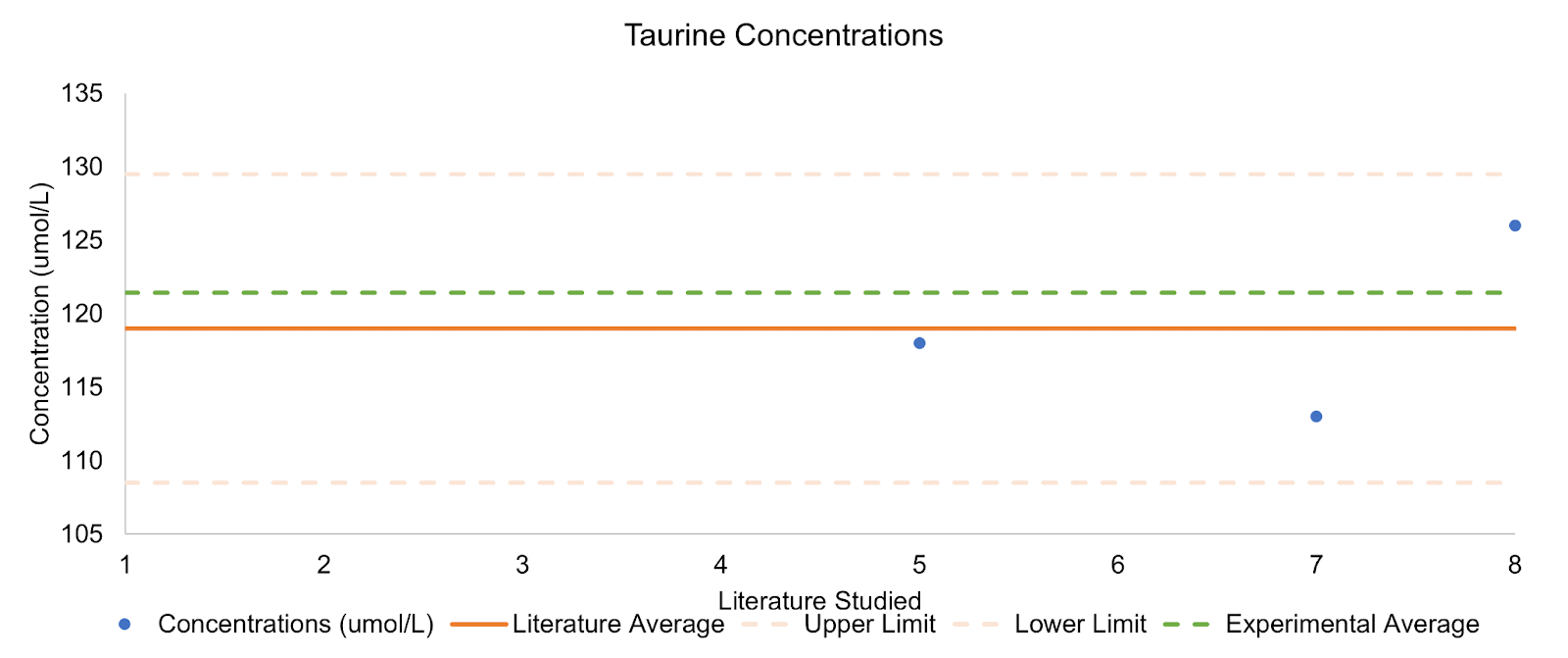


**Figure 19** The distribution of taurine concentrations as they appear in the literature and through experimental values. Experimental values and literature averages are statistically equivalent (p=0.34, a=0.05)


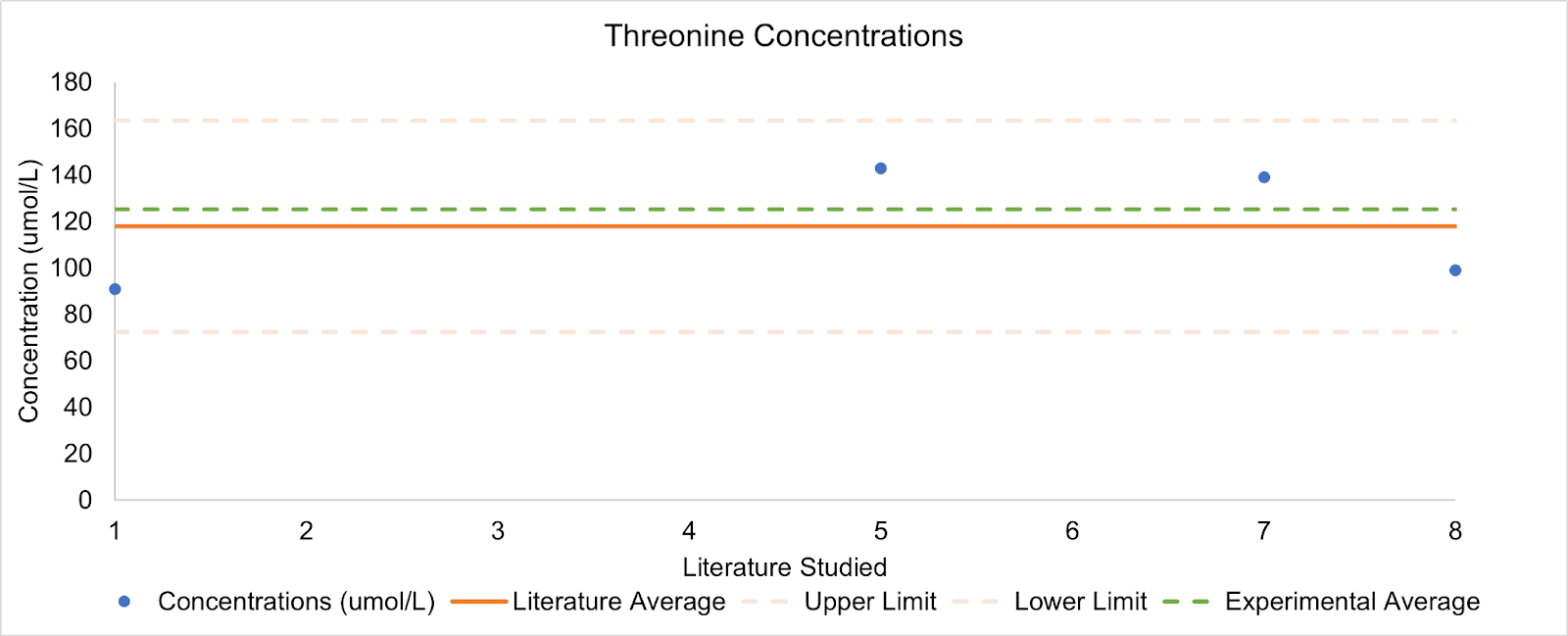


**Figure 20** The distribution of threonine concentrations as they appear in the literature and through experimental values. Experimental values and literature averages are statistically equivalent (p=0.36, a=0.05)


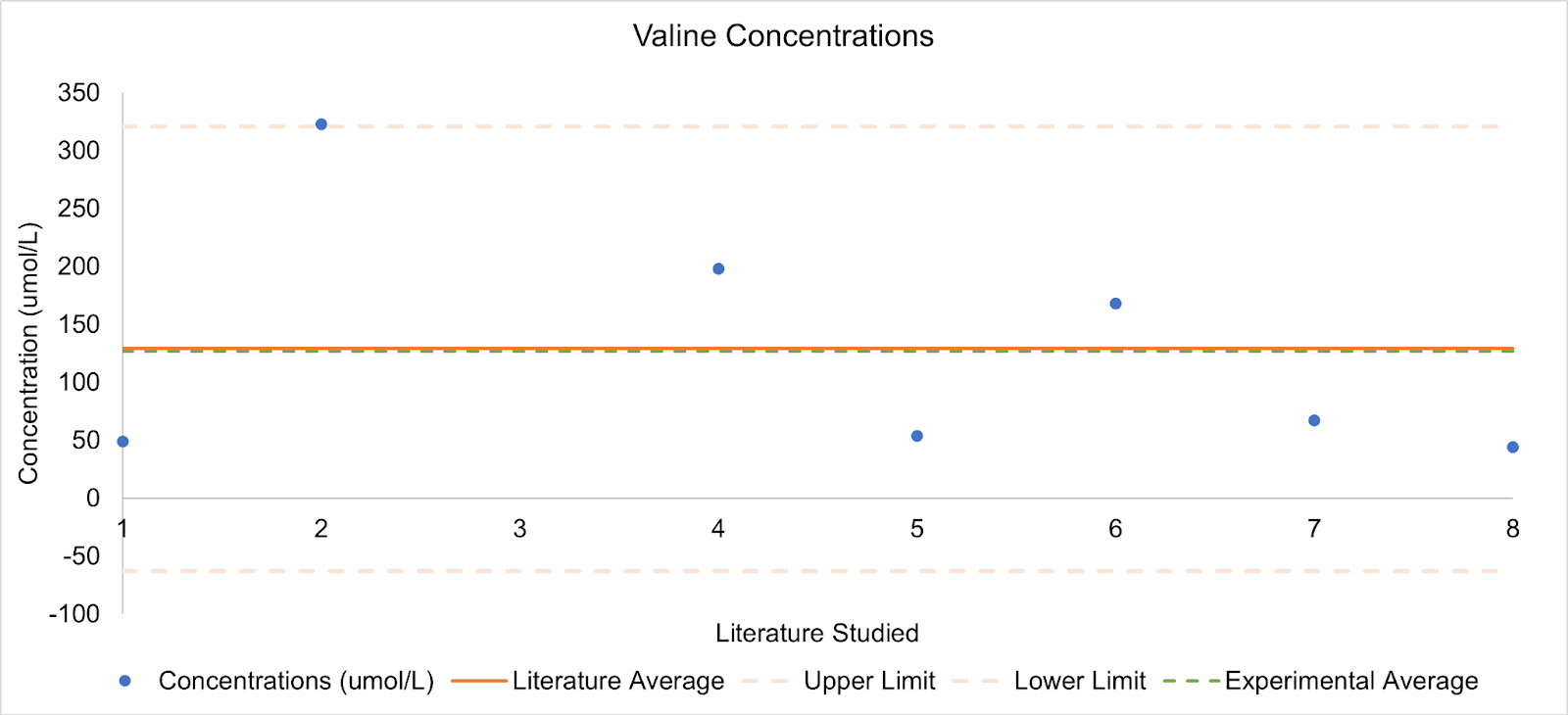


**Figure 21** The distribution of valine concentrations as they appear in the literature and through experimental values. Experimental values and literature averages are statistically equivalent (p=0.48, a=0.05)


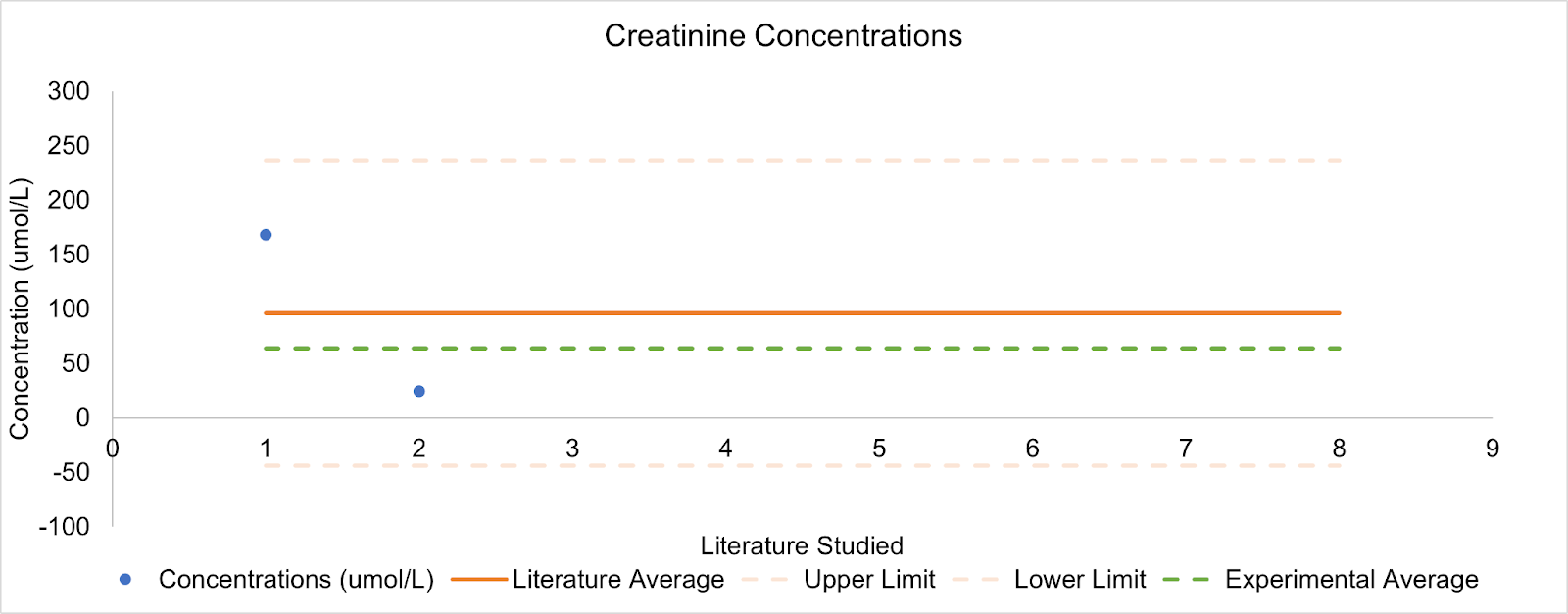


**Figure 22** The distribution of creatinine concentrations as they appear in the literature and through experimental values. Experimental values and literature averages are statistically equivalent (p=0.37, a=0.05)


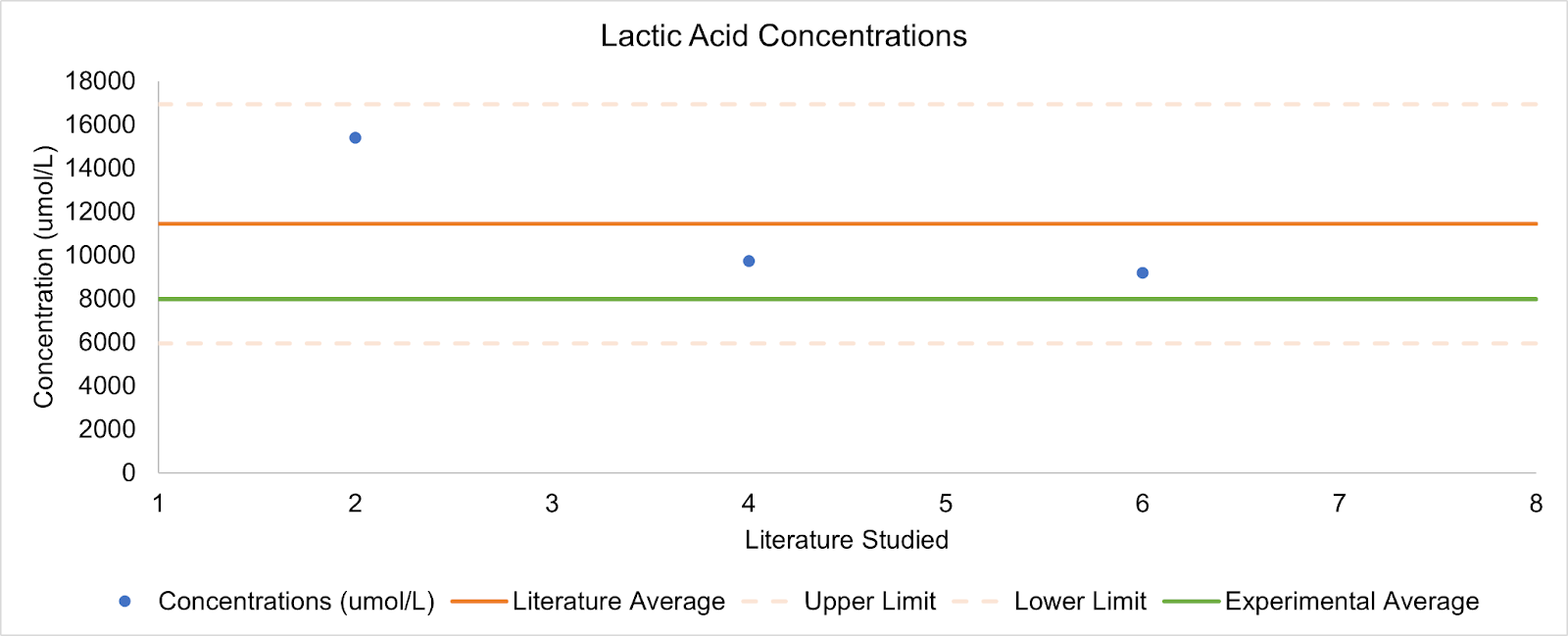


**Figure 23** The distribution of lactic acid concentrations as they appear in the literature and through experimental values. Experimental values and literature averages are statistically equivalent (p=0.11, a=0.05)
